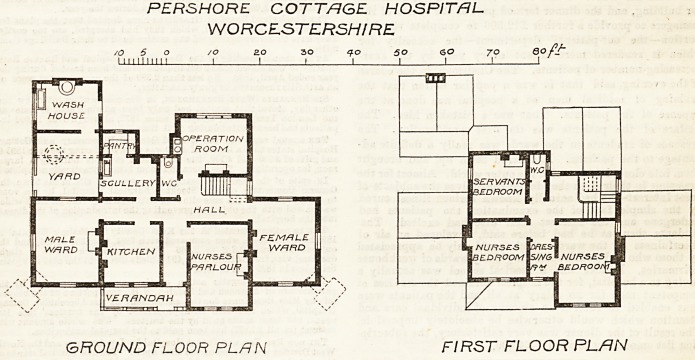# Hospital Construction

**Published:** 1896-06-27

**Authors:** 


### HOSPITAL CONSTRUCTION.

### PERSHORE COTTAGE HOSPITAL, WORCESTERSHIRE.

These plans have been prepared by Mr. J. H. Williams, of Worcester. There is no indication of the aspects of the building. It consists of two wards, 18 feet by 14 feet, one for males and one for females; an operating-room, 14 feet square; a nurses' parlour, a kitchen, scullery, w.c., pantry, and washhouse. The upper floor, extending over a portion of the building—not over the wards or operating-room—contains a servant's bed-room, two nurses' bed-rooms, a very small dressing-room, and a w.c.

The principal entrance is between the nurses' parlour and the kitchen, and leads into a passage, with the wards at either end. This passage derives some light from the staircase window, but is not otherwise lighted, and would be dark. The kitchen and scullery are on opposite sides of this passage—a very bad and inconvenient arrangement. No bath-room is provided for either patients or nurses. No duty-room for nurses seems to have been thought of, and the sanitary appliances seen ill-placed and insufficient.

The control over the male ward seems to have been lost sight of, the nurses' room being dissociated from it. The verandah overhanging the kitchen window can add nothing to its cheerfulness. The number and intended position of the beds in the wards is not indicated, and, with the arrangement of windows shown on the plans, might be a matter of difficulty.

The omission of reasonable and necessary sanitary arrangements for the inmates, and the awkward disposition of the kitchen and offices, are serious drawbacks in the plans. The w.c. close to the entrance to the operating-room is also objectionable, and the position of the operating-room itself not the best that could have been chosen.

## Figures and Tables

**Figure f1:**